# CT findings of the temporal bone in CHARGE syndrome: aspects of importance in cochlear implant surgery

**DOI:** 10.1007/s00405-016-4141-z

**Published:** 2016-06-20

**Authors:** A. C. Vesseur, B. M. Verbist, H. E. Westerlaan, F. J. J. Kloostra, R. J. C. Admiraal, C. M. A. van Ravenswaaij-Arts, R. H. Free, E. A. M. Mylanus

**Affiliations:** 1 Department of ENT, Radboud University Medical Centre, Postbus 9101, 6500 HB Nijmegen, Netherlands; 2 Department of Radiology, Radboud University Medical Centre, Postbus 9101, 6500 HB Nijmegen, Netherlands; 3 Department of Radiology, Leiden University Medical Centre, Postbus 9600, 2300 RC Leiden, Netherlands; 4 Department of Radiology, University Medical Centre Groningen, University of Groningen, Postbus 30.001, 9700 RB Groningen, Netherlands; 5 Department of ENT, University Medical Centre Groningen, University of Groningen, Postbus 30.001, 9700 RB Groningen, Netherlands; 6 Department of Genetics, University Medical Centre Groningen, University of Groningen, Postbus 30.001, 9700 RB Groningen, Netherlands

**Keywords:** CHARGE syndrome, Cochlear implant, Anatomy, Genetics, Temporal bone

## Abstract

To provide an overview of anomalies of the temporal bone in CHARGE syndrome relevant to cochlear implantation (CI), anatomical structures of the temporal bone and the respective genotypes were analysed. In this retrospective study, 42 CTs of the temporal bone of 42 patients with CHARGE syndrome were reviewed in consensus by two head-and-neck radiologists and two otological surgeons. Anatomical structures of the temporal bone were evaluated and correlated with genetic data. Abnormalities that might affect CI surgery were seen, such as a vascular structure, a petrosquamosal sinus (13 %), an underdeveloped mastoid (8 %) and an aberrant course of the facial nerve crossing the round window (9 %) and/or the promontory (18 %). The appearance of the inner ear varied widely: in 77 % of patients all semicircular canals were absent and the cochlea varied from normal to hypoplastic. A stenotic cochlear aperture was observed in 37 %. The middle ear was often affected with a stenotic round (14 %) or oval window (71 %). More anomalies were observed in patients with truncating mutations than with non-truncating mutations. Temporal bone findings in CHARGE syndrome vary widely. Vascular variants, aberrant route of the facial nerve, an underdeveloped mastoid, aplasia of the semicircular canals, and stenotic round window may complicate cochlear implantation.

## Introduction

The criteria for the clinical diagnosis of CHARGE syndrome (MIM, Mendelian Inheritance in Man, 214800), have been defined by Blake et al. and Verloes [1, 2]. CHARGE syndrome is an acronym of Coloboma, Heart disease, choanal Atresia, Retardation, Genital hypoplasia, and Ear anomalies. Organ involvement and severity is highly variable amongst affected patients. A major criterion includes the condition of the temporal bone, which may be hypoplastic or show an absence of the semicircular canals, according to Verloes. Anomalies are seen in the external, middle and inner ear, such as the typically low-set, cup-shaped ears, ossicular malformations, an aberrant course of the facial nerve, hypoplastic internal auditory canal, and an abnormally developed cochlea. Some of these malformations can cause hearing loss: 60–90 % of patients with CHARGE syndrome have moderate to severe hearing loss due to conductive, sensorineural or mixed defects. In most patients, hearing loss can be partially compensated with hearing aids. When hearing aids do not have the desired outcome due to the presence of profound to severe hearing loss, cochlear implantation may be considered. If cognitive disabilities, developmental and behavioural problems do not preclude cochlear implantation, a thorough assessment of the temporal bone anatomy is necessary. Anatomical alterations pose additional surgical risks during the implantation, by hampering the surgical approach to the cochlea or the insertion of the electrode array into the cochlea, and they may influence the surgical results in terms of speech perception.

In 2004, the causative gene for CHARGE syndrome was identified as *CHD7* on chromosome 8q12.1 [3]. Since then, 528 different mutations of the gene have been described, but no clear genotype-phenotype correlation could be recognized (www.CHD7.org) [4]. In the *CHD7* mutation positive patients, the most common clinical findings were temporal bone anomalies (98 %), external ear malformations (91 %), and hearing loss (89 %) [5].

The main goal of this retrospective study was to analyse the presence of the anomalies of the temporal bone and the cochlear nerve in patients with CHARGE syndrome and their potential impact on cochlear implant surgery planning. The secondary goal was to study possible genotype-phenotype correlations.

## Materials and methods

We collected analogue and digital CT studies of the temporal bone of patients attending the Dutch CHARGE center of expertise (University Medical Center Groningen, the Netherlands), after obtaining written informed consent from all patients or their legal representatives.

All patients had molecularly confirmed CHARGE syndrome, or clinically typical CHARGE syndrome according to the Blake or Verloes criteria (Tables 1, 4) except for one patient with atypical CHARGE syndrome (patient 12), because the parents did not wish further investigation [1, 2].

**Table 1:** Characteristics of CHARGE syndrome

a. Major and minor signs of CHARGE syndrome [2]
Major signs
Coloboma (iris or choroid, with or without microphthalmia)
Atresia of choanae
Hypoplastic semicircular canals
Minor signs
Rhombencephalic dysfunction (brainstem dysfunctions, cranial nerve VII to XII palsies and neurosensory deafness)
Hypothalamo-hypophyseal dysfunction (including GH and gonadotrophin deficiencies)
Abnormal middle or external ear
Malformation of mediastinal organs (heart, oesophagus)
Intellectual disability
b. Definition of typical, atypical, and partial CHARGE syndrome [2]
Typical CHARGE syndrome
3 major signs
2/3 major signs + 2/5 minor signs
Partial/incomplete CHARGE
2/3 major signs + 1/5 minor signs
Atypical CHARGE
2/3 major signs + 0/5 minor signs
1/3 major signs + 3/5 minors signs

**Table 2:** Ear structure observations

Structure on CT	Normal	Abnormal	UTI
Pneumatisation mastoid	Good	No cells	UTI
	73 (87 %)	7 (8 %)	4 (5 %)
Middle ear cavity size	Normal	Small/large	UTI
	84 (100 %)	0	0
Jugular bulb	Normal	High	UTI
	59 (70 %)	23 (27 %)	2 (2 %)
Emissary veins	Total	>1 mm	PSS
	28 (33 %)	25 (30 %)	11 (13 %)
Windows	Present	Absent/stenotic	UTI
Oval	22 (26 %)	60 (71 %)	2 (2 %)
Round	70 (83 %)	12 (14 %)	2 (2 %)
Ossicles	Normal	Dysplastic	UTI
Malleus	83 (99 %)	1 (1 %)	0
Incus	75 (89 %)	9 (11 %)	0
Stapes	27 (32 %)	42 (50 %)	13 (15 %)
Facial nerve	Normal	Aberrant course	UTI
Tympanic	54 (64 %)	24 (29 %)	6 (7 %)
Mastoid	70 (83 %)	6 (7 %)	8 (10 %)
Vestibular aqueduct	Normal	Aberrant course	UTI
	12 (14 %)	57 (68 %)	12 (14 %)
Cochlear apertura	Normal	Stenotic	UTI
	51 (61 %)	31 (37 %)	2 (2, 4 %)
SCC	Normal	Aplastic	Dysplastic
	2 (2 %)	65 (77 %)	17 (20 %)

Number of ears: 84

*UTI* unable to identify; *PPS* persistent petrosquamosal sinus, *SCC* semicircular canals

**Table 3:** Ear structure measurements

Structure	Mean (mm)	Median (mm)	SD (mm)	Max (mm)	Min (mm)	UTI (ears)
Mastoid						
AP-size	10.6	11.0	3.2	19.4	5.0	21
LM-size	7.9	7.0	6.2	40.0	1.2	22
Vestibulum						
Length	4.7	4.7	1.0	9.0	2.9	0
Width	2.3	2.3	0.6	5.0	1.0	1.0
VA diameter^a^						
	0.7	0.6	0.3	1.9	0.1	11
IAC						
	3.6	3.5	0.9	7.0	2.0	0

Number of ears: 84

^a^only digital scans (*n* = 58)

*AP* anterior-posterior, *LM* lateral-medial, *VA* vestibular aqueduct, *IAC* internal auditory canal, *SD* standard deviation, *UTI* unable to identify

**Table 4:** Mutations

Patient no.	Mutation	Mutation type	Blake/Verloes criteria
1	Nonsense	Truncating	Positive
2	Missense	Non-truncating	Negative
3	Nonsense	Truncating	Positive
4	Nonsense	Truncating	Positive
5	Splice-site	Non-truncating	Negative
6	Splice-site	Non-truncating	Positive
7	Nonsense	Truncating	Positive
8	Missense	Non-truncating	Positive
9	Splice-site	Non-truncating	Atypical
10	Splice-site	non-truncating	Atypical
11	Missense	Non-truncating	Atypical
12	No mutation		Atypical
13	Missense	Non-truncating	Negative
14	Frameshift	Truncating	Positive
15	Frameshift	Truncating	Positive
16	Nonsense	Truncating	Positive
17	Nonsense	Truncating	Positive
18	Missense	Non-truncating	Positive
19	Nonsense	Truncating	Positive
20	Frameshift	Truncating	Positive
21	Splice-site	Non-truncating	Negative
22	Nonsense	Truncating	Positive
23	Splice-site	Non-truncating	Positive
24	Nonsense	Truncating	positive
25	Frameshift	Truncating	Positive
26	Frameshift	Truncating	Positive
27	Frameshift	Truncating	Positive
28	Missense	Non-truncating	Positive
29	Frameshift	Truncating	Positive
30	UV missense		Atypical
31	Frameshift	Truncating	Partial
32	Nonsense	Truncating	Positive
33	Splice-site	Non-truncating	Positive
34	Missense	Non-truncating	Positive
35	Splice-site	Non-truncating	Atypical
36	Nonsense	Truncating	Positive
37	Nonsense	Truncating	Positive
38	Frameshift	Truncating	Positive
39	Nonsense	Truncating	Positive
40	Frameshift	Truncating	Positive
41	Nonsense	Truncating	Atypical
42	Deletion	Truncating	Positive

*UV* unknown variant

**Table 5:** Distribution of cochlear types for different types of mutations

Cochlear type	Normal	IPII^a^	Hypoplasia type III	Hypoplasia type IV
Total	49 (61.2 %)	7 (8.3 %)	3 (3.8 %)	21 (26.3 %)
Truncating mutations	27 (33.8 %)	6 (7.5 %)	3 (3.8 %)	14 (17.5 %)
Non-truncating mutations	22 (27.5 %)	1 (1.3 %)	0	7 (8.8 %)

Number of ears: 80 (patients 12 and 30 excluded)

^a^Incomplete partition type II without enlarged vestibular aqueduct or dilated vestibulum; *P* = 0.194 (χ^2^)

**Table 6:** Distribution of semicircular canal malformations for different types of mutations

Defect	SCC normal	PSCC dysplasia	SSCC dysplasia	PSCC + LSCC dysplasia	SCCC + LSCCC dysplasia	All aplastic	All dysplastic
Total	2 (2.5 %)	4 (5.0 %)	5 (6.3 %)	1 (1.3 %)	2 (2.5 %)	63 (78.8 %)	3 (3.8 %)
Truncating	0	0	3 (3.8 %)	0	0	46 (57.5 %)	1 (1.3 %)
Non-truncating	2 (2.5 %)	4 (5.0 %)	2 (2.5 %)	1 (1.3 %)	2 (2.5 %)	17 (21.3 %)	2 (2.5 %)

Number of ears: 80 (patients 12 and 30 excluded); *P* = 0.004 (χ^2^)

*SCC* semicircular canals, *PSCC* posterior semicircular canal, *SSCC* superior semicircular canal, *LSCC* lateral semicircular canal

**Table 7:** Surgical challenges in cochlear implantation

Surgical step	Structure on CT	Anomaly	Surgical challenge
Mastoidectomy	Mastoid	Underdeveloped	Reduced access to the middle ear
	Vascular structures	Large emissary vein	Unexpected bleeding
		PSS	Bleeding, reduced mastoid size
	Semicircular canals	Aplasia	Loss of landmark
Post. tympanotomy	Facial nerve	More medial route	Facilitates entrance to the middle ear
Cochleostomy	Ossicles	Dysplasia	Obstructed vision by the incus
	Facial nerve	Aberrant route	Impedes cochleostomy
	Windows	Round window stenosis	Choosing optimal side for cochleostomy
	Jugular bulb	High	Preparing cochleostomy
Insertion	Cochlea	Aplasia	Insertion

*Post.* posterior, *PSS* petrosquamosal sinus

**Table 8:** Combinations of anomalies often seen

Anomaly	In combination with	Number of patients (percentage of all patients)
Absent RW	Absent OW	12 (14.3 %)
Stenotic OW	Dysplastic stapes	23 (27.3 %)
Aberrant tympanic portion facial nerve	Dysplastic stapes	24 (28.6 %)
Total SCC hypoplasia	OW stenosis	21 (25.0 %)
Aberrant VA	SCC hypoplasia with SCA	14 (16.7 %)
	SCC hypoplasia without SCA	14 (16.7 %)

*OW* oval window, *RW* round window, *SCC* semicircular canals, *SCA* stenotic cochlear aperture, *VA* vestibular aqueduct

The patients were investigated in different time periods and in different Dutch hospitals, so the scans were made with different scanner types and variable scan parameters.

We evaluated CTs of 84 ears of 42 patients (22 male, 20 female) with CHARGE syndrome (29 digital and 13 analogue scans). The scans were performed between 1996– 2010. The mean age of the patients at the time of scanning was 6.4 years, median 2.5 years (SD 9.8; min 0, max 47 years).

### Imaging analysis

**Fig. 1:**
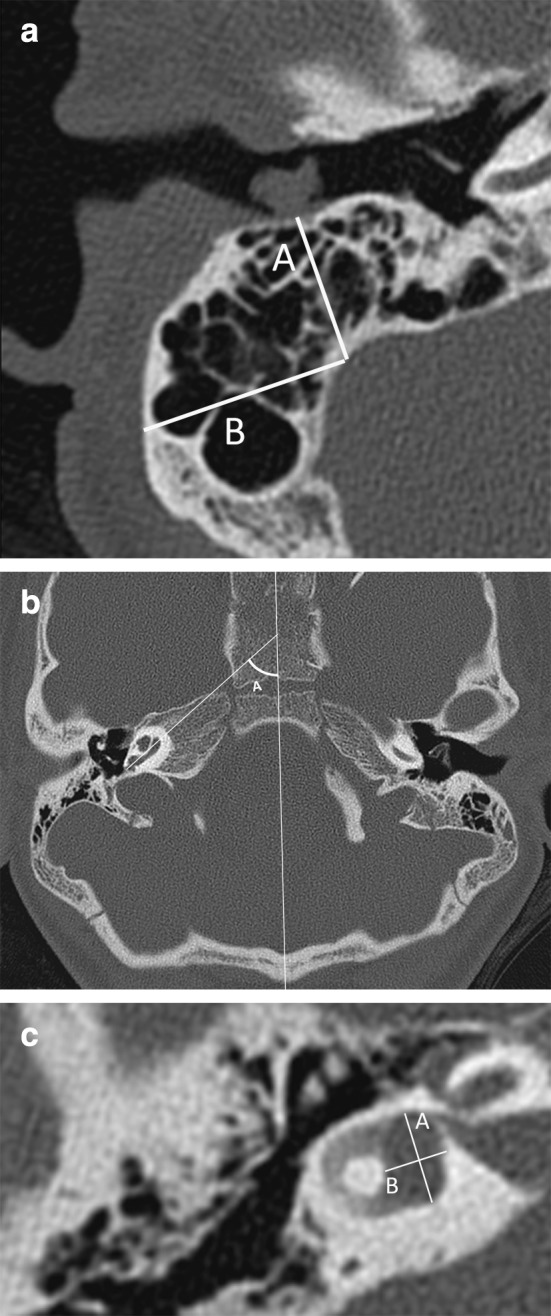
Measurements in axial CT images. **a** Mastoid size *A* anterior-posterior (AP) size, measured in the middle of the external meatus (cranial/caudal) as the minimal distance from the external meatus to sigmoid sinus *B* lateral-medial (LM) size, distance between outer cortex and sigmoid sinus, measured perpendicular to A. **b** Angle cochlear basal turn. **c** Vestibulum size *A* longitudinal extension, *B* transversal diameter (right ear)

All imaging studies were evaluated by four observers (two head-and-neck radiologists with 13 and 7 years of experience and two otorhinolaryngologists with 10 and 15 years of experience), who then met to reach a consensus opinion. The reviewers had no access to patients’ names or their clinical information. Each ear was evaluated separately, in axial and coronal planes, if available. The anatomic structures and normative measures determined are presented in Table 9 in “Appendix” and Fig. 1.

**Table 9:** Radiologic criteria for the os petrosum in patients with CHARGE syndrome

Mastoid and vascular structures	
Mastoid	Observation: pneumatisation of one or more cells Measurement: AP size: minimal distance from external meatus wall to sigmoid sinus taken at the middle of the meatus in the axial plane Measurement: LM size: minimal distance from cortex to sinus at the most anterior border of the sinus perpendicular to the mastoid AP size
Emissary veins	Observation: emissary veins through temporal squama, persistent petro-squamosal sinus [13] Measurement: >1 and <1 mm
Jugular bulb	Observation: high if at the level or cranial of the round window in axial plane
Middle ear	
Ossicles	Observation: dysplasia
Windows	Observation: stenotic
Facial nerve	Observation: normal with present SCC: in transverse plane caudal of the LSCC and lateral and superior of the oval window. In coronal plane lateral and medial of the SCC Normal with absent SCC: coronal plane cranial of the oval window, posterior of the axis of the basal turn of the cochlea at the level of the anterior rim of the round window
Cochlear vestibular system	
Cochlea	Measurement: angle basal turn and midline skull (54.6 degrees (range 46.8°–63.8°; standard deviation, 3.5) [24] Observation: dysplasia [31] with separate judgment of modiolus.
SCC	Absent, dysplastic, normal
Vestibulum	Measurement length: maximum longitudinal extension, width maximum transversal diameter, perpendicular to the length [22]. (normal (6.18–6.42) × (3.44–3.59) mm, interval)
Vestibular aqueduct	Measurement: diameter at midpoint (normal 1.5–2 mm) Observation: course
IAC	Measurement: Midline in axial plane(normal 2–8 mm)
Cochlear aperture	Observation: present or bony stenosis
Nerves in IAC	Observation on MRI: normal, hypoplastic or aplastic

*AP* anterior-posterior, *LM* lateral-medial, *SCC* semicircular canal, *LSCC* lateral semicircular canal, *IAC* internal auditory canal

All the scans were analysed as extensively as possible, using a standardized form (Table 10 in “Appendix”) compiled specifically for this study. Items that could not be analysed, e.g. due to a missing coronal plane or to slice thickness, were scored as ‘unable to identify’ (UTI).

**Table 10:**
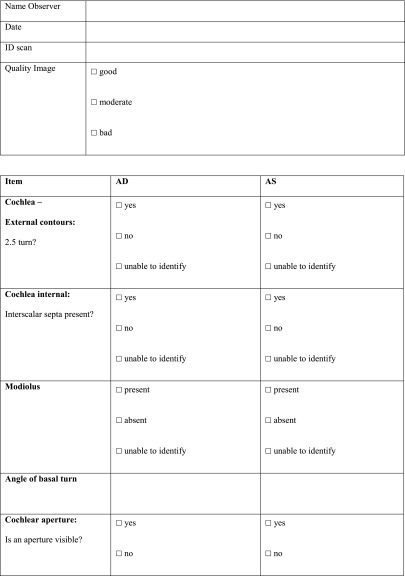

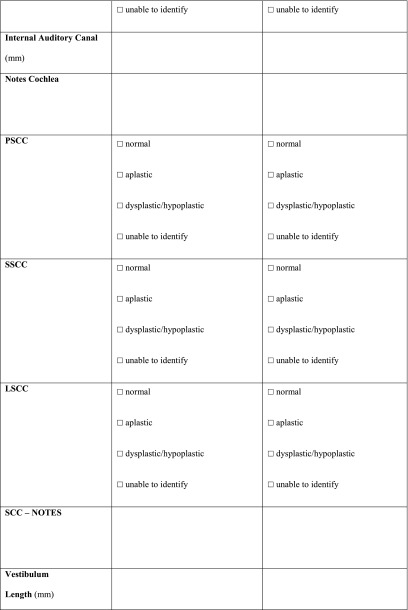

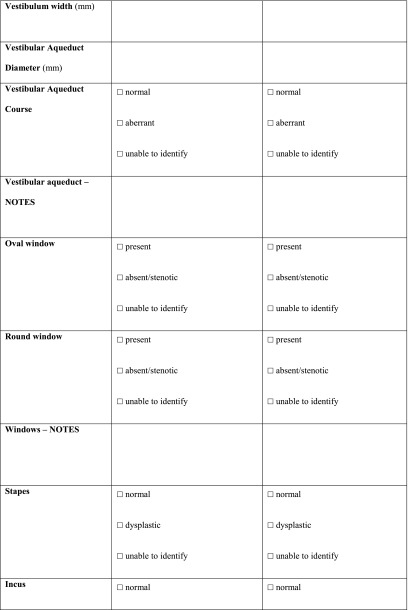

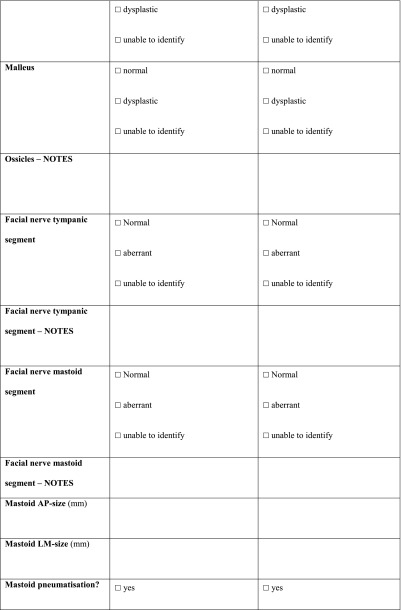

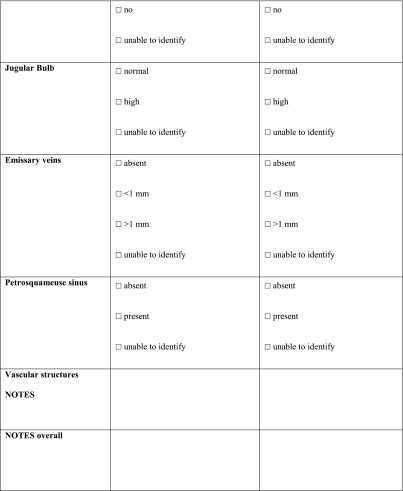
Radiologic set of criteria for CT scan

*PSCC* posterior semicircular canal, *SSCC* superior semicircular canal, *LSCC* lateral semicircular canal, *SCC* semicircular canal, *AP* anterior-posterior, *LM* lateral-medial

The digital scans were analysed on a viewing station (IMPAX, Apache Software Foundation, Version 2.0, January 2004). Measurements were obtained in millimetre (to two decimal places) with electronic calipers provided by the pacs-system. Analogue films were evaluated on an illuminated view box and measurements were performed with an analogue ruler. If a structure could not be properly assessed, it was scored as ‘unable to identify’ (UTI).

SPSS 20 was used to collect all data and perform statistical analyses. We used the* χ* ^2^ test to test for significant correlations.

### CHD7 analysis

The results of *CHD7* analysis were already known for all but one (patient 12) patient. The analyses were performed on DNA isolated from peripheral blood cells according to standard procedures. The 37 coding exons of *CHD7* (exons 2–38, RefSeq NM_017780.02) and their flanking intron sequences were amplified by PCR and sequenced as described earlier [3]. If no mutations were identified, *CHD7* was screened for whole exon deletions and duplications by multiplex ligation-dependent probe amplification (MLPA) using a commercially available set of probes: the SALSA P201 kit (MRC-Holland, Amsterdam, the Netherlands; http://www.mrc-holland.com) [6].

Nonsense and frameshift mutations and whole-gene or whole-exome deletions were categorised as truncating mutations, while missense and splice site mutations were categorised as non-truncating.

## Results

### Mastoid and vascular structures (Tables 2, 3)

**Fig. 2:**
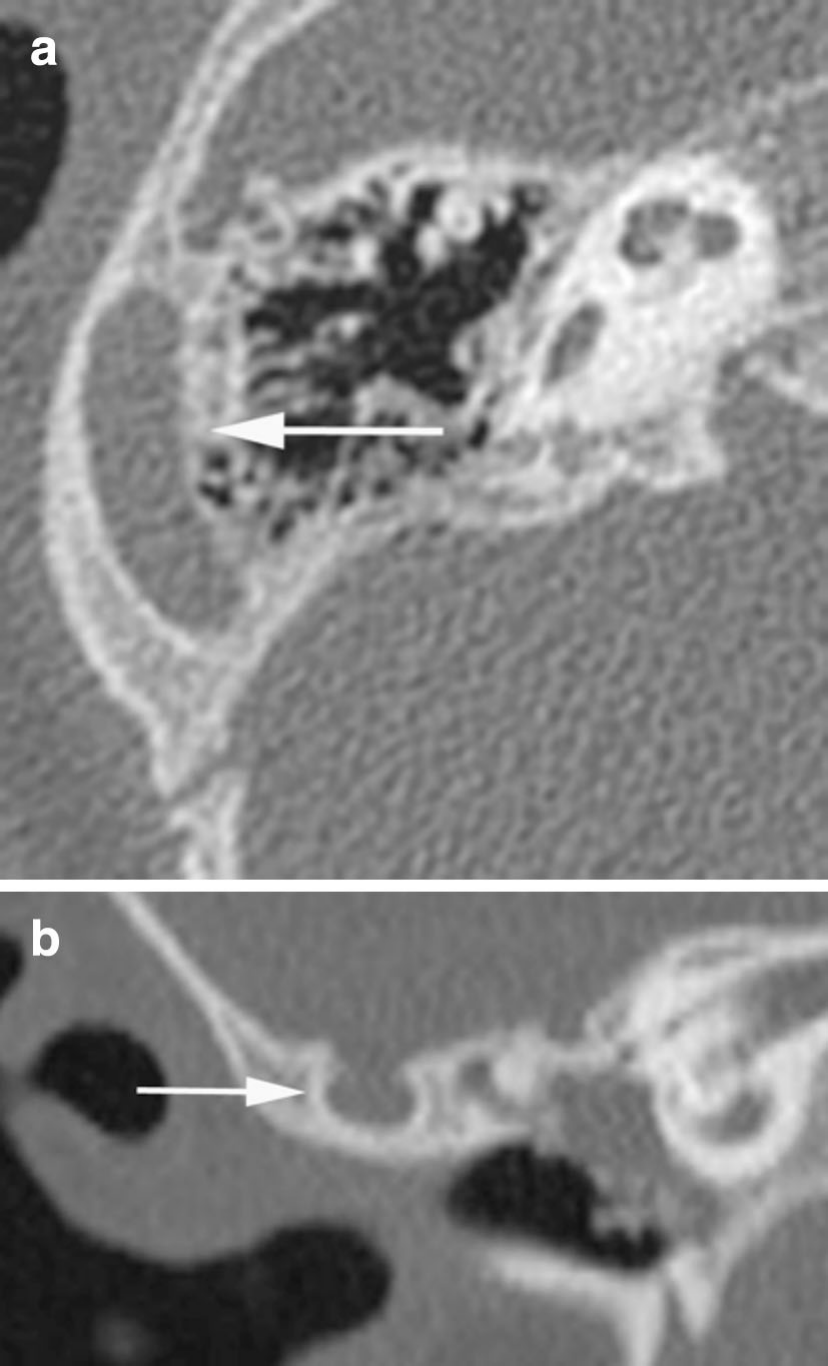
Petrosquamosal sinus. Axial (**a**) and coronal (**b**) CT image of a right ear showing this emissary vein coursing along the lateral superior surface of the temporal bone. The petrosquamosal sinus originates at the transverse sinus and drains either into the retromandibular vein or the pterygoid venous plexus

The first part of a cochlear implantation, the mastoidectomy, can be challenging in an underdeveloped mastoid. The AP-size (anterior-posterior) and the LM-size (lateral-medial) of the mastoid could not be measured in 21 ears (25 %), because of a hardly developed mastoid or moderate quality of the scan. These patients were particularly young (median age 5 years, mean 8.5 years, 22 % <1-year-old). In 25 ears (29.8 %), an emissary vein with a diameter larger than 1 mm was present (Fig. 2a, b).

### Middle ear (windows/ossicles/facial nerve) (Tables 2, 3)

Middle ear anomalies can be a challenge in preparing and making the cochleostomy. The size of the middle ear cavity was within normal limits in all patients and thus will not pose a problem in surgery. Overall, there was an aberrant course of the tympanic part of the facial nerve crossing the promontory in 16 ears (19.0 % of the total number of ears) and in eight ears also the round window (9.5 % of the total number of ears). The aberrant mastoidal portion of the facial nerve seemed to run more medially than normal in four ears. The windows and ossicles were difficult to assess due either to otitis media or to the moderate quality of the scan in 20 patients (23.8 %).

**Fig. 3:**
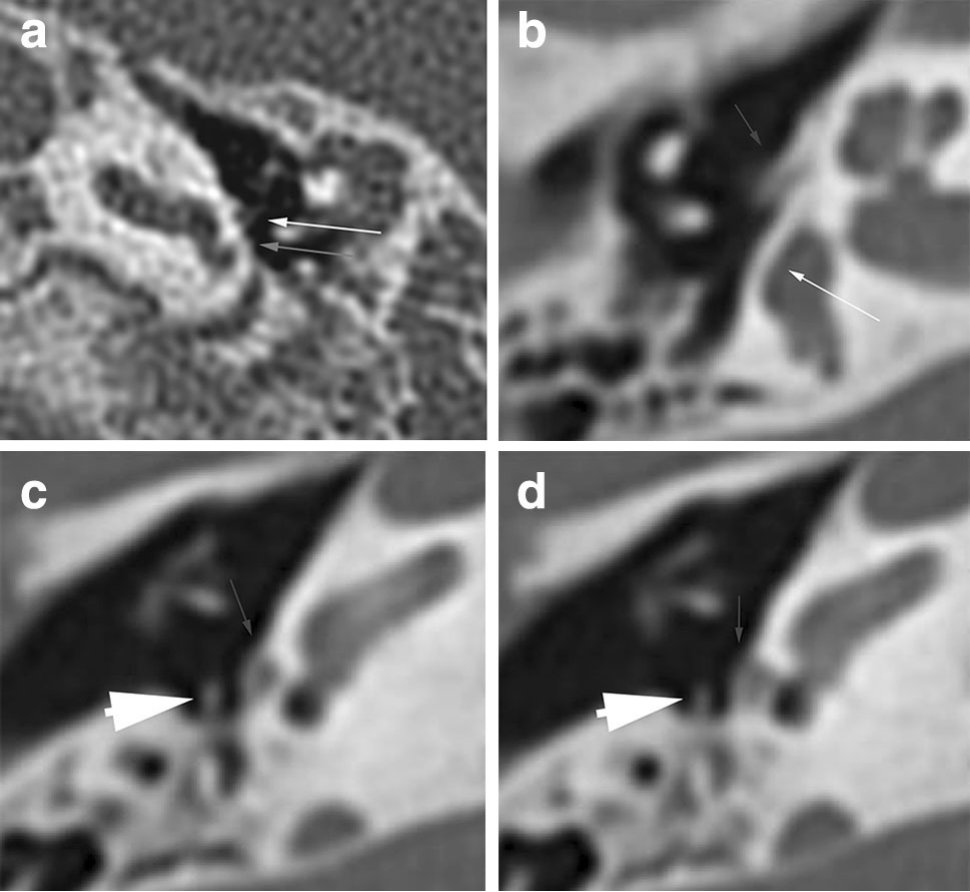
Examples of window stenosis. **a** Axial CT image showing stenosis of the round window niche (*grey arrow*) in a left ear. Note also dysplastic stapes on the promontory (*white arrow*). **b**–**d** Axial CT images showing atresia of the oval window (*thin arrow*), aberrant course of the facial nerve crossing the round window (*arrowhead*) and a dysplastic stapes positioned at the sinus tympani (*thick arrow*). Note aplasia of the semicircular canals

In 43 ears (51.2 %) with a stenotic oval window, the stapes was not identifiable or dysplastic, either presenting as a monopod stapes (one ear), or displaced on the promontory or into the middle ear cavity (six ears) (Fig. 3).

### Cochleovestibular system (Tables 5, 6)

**Fig. 4:**
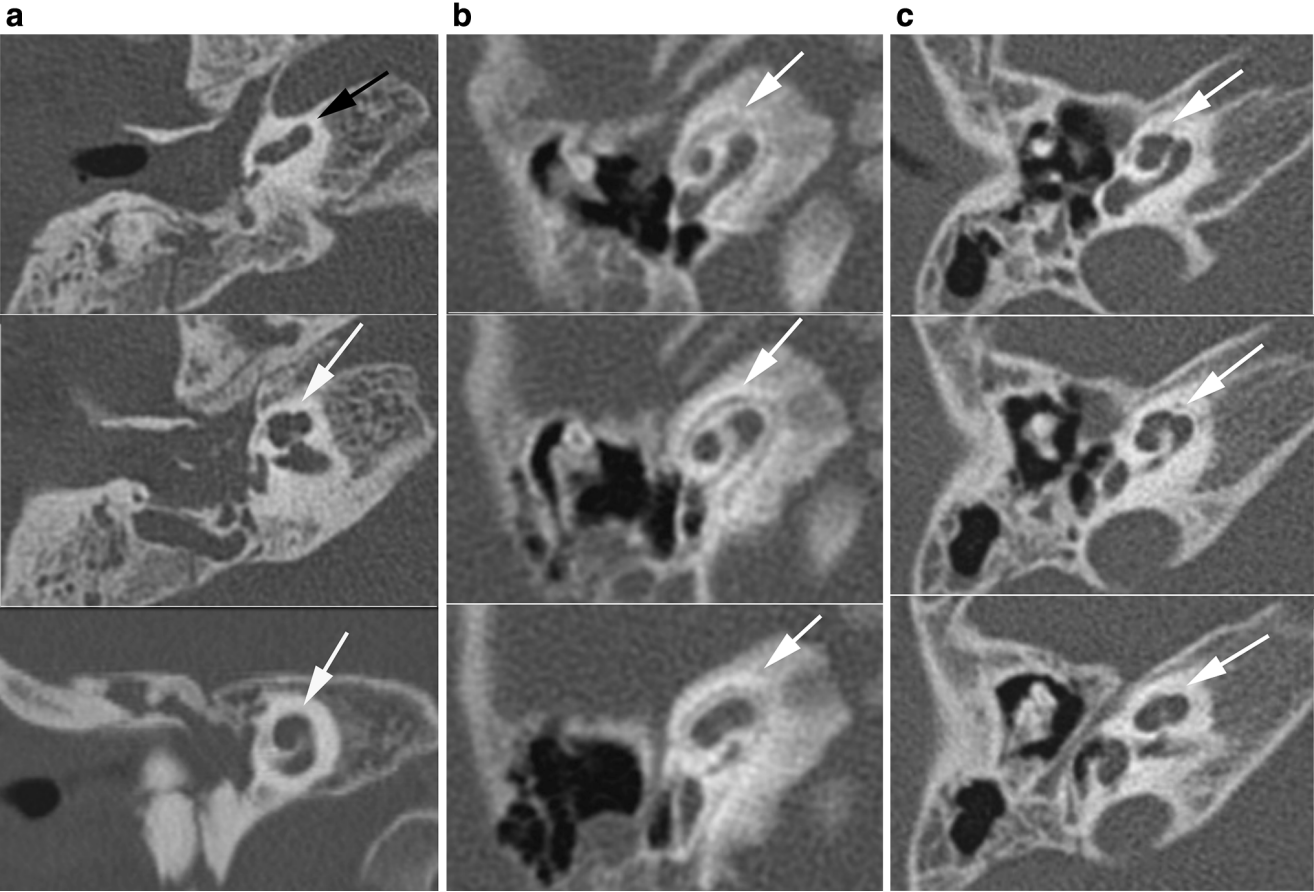
Range of abnormalities of the cochlea seen in axial CT images. **a** Incomplete partitioning type II: normal development of the basal turn, but fusion of the second and apical turn seen in axial and coronal planes. **b** Hypoplasia type III: cochlea with less than 2 turns. **c** Cochlea type ‘IV’: the basal, second and apical turns are present, but the second turn seems shortened, giving the cochlea an asymmetric, flattened appearance

Abnormalities of the cochlea can complicate the insertion of the electrode array. Table 5 shows the distribution of cochlear type, omitting patient 12 (who had no mutation found and normal cochleas), and patient 30 (who had a UV missense mutation, one normal cochlea and one cochlear hypoplasia type IV). In 32 (38.1 %) ears, an abnormal cochlea was seen. The ears with an incomplete partition type II (IPII) deformity of the cochlea did not show an enlarged vestibular aqueduct or dilated vestibulum. In 22 (26.2 %) ears, the cochlea appeared abnormal, but the type of dysplasia could not be determined according to Sennaroglu’s classification (Fig. 4a–c). In these cochleae, the second turn seemed not to have developed fully, but the apex and basal turn were normal, with normal presence of interscalar septae and spiral osseous lamina, and in all but two of them the modiolus was normal. We will refer to this as hypoplasia type IV.

The angle of the basal turn was only measured on the digital scans; the mean was 57° (SD 6.3), with a range from 43.5° to 78.6°. The mean age of this group was 3.7 years.

**Fig. 5:**
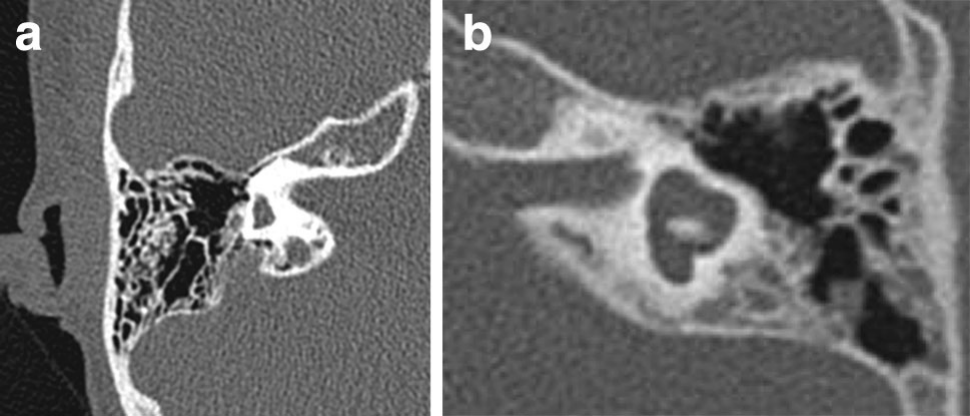
Range of abnormalities of the vestibular system seen in axial CT images. **a** Aplasia of the semicircular canals in a right ear. **b** Dysplasia of the vestibule and semicircular canals in a left ear, with a malformed vestibule, shortened and dilated horizontal semicircular canal with small bony island, incomplete formation and dilatation of the posterior semicircular canal

Table 6 shows the distribution of SCC malformations excluding patient 12 (no mutation found and dysplasia of LSCC bilaterally) and patient 30 (UV missense mutation and total aplasia of SCC bilaterally). Aplasia of all SCCs was seen in 65 ears (77.3 %), while dysplasia of one or all SCCs was seen in 17 ears (20.2 %) and ranged from the strongly reduced development of one canal, like a bud, to just one affected canal (while the other two were present and normal) (Fig. 5a, b). In ears with a solitary canal aplasia or dysplasia, it was the lateral semicircular canal that was most often affected. If the superior semicircular canal was dysplastic, the lateral and posterior semicircular canals were absent.

Generally, the vestibulum was smaller than normal, both in length and width. The aberrant vestibular aqueducts had a course mainly in a perpendicular line from the vestibulum to the posterior fossa. If SCCs were absent, the aqueduct showed a more medial course than when they were severe or mild dysplastic. We found one ear with a large vestibular aqueduct (1.9 mm diameter), but normally developed cochlea.

### Cochlear aperture and inner ear (Table 2)

**Fig. 6:**
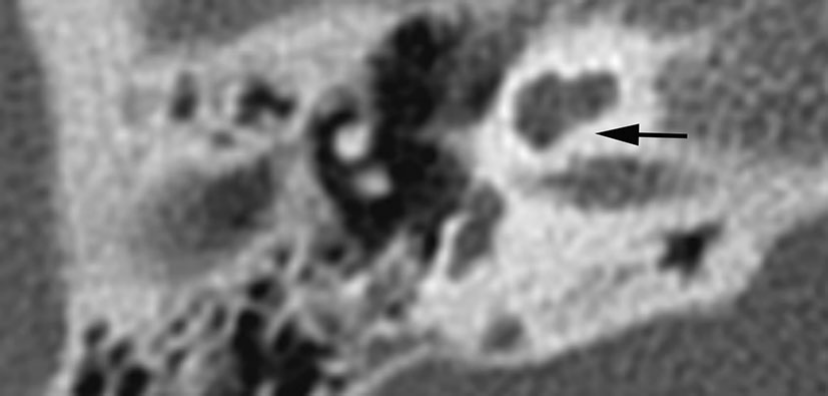
Cochlear aperture—axial CT image shows a lacking cochlear aperture

In 13 of 31 ears with a stenotic aperture, the cochlea was abnormal (one incomplete partitioning type II, three hypoplasia type III, and nine type IV) (Table 2; Fig. 6).

### Surgical challenges

**Fig. 7:**
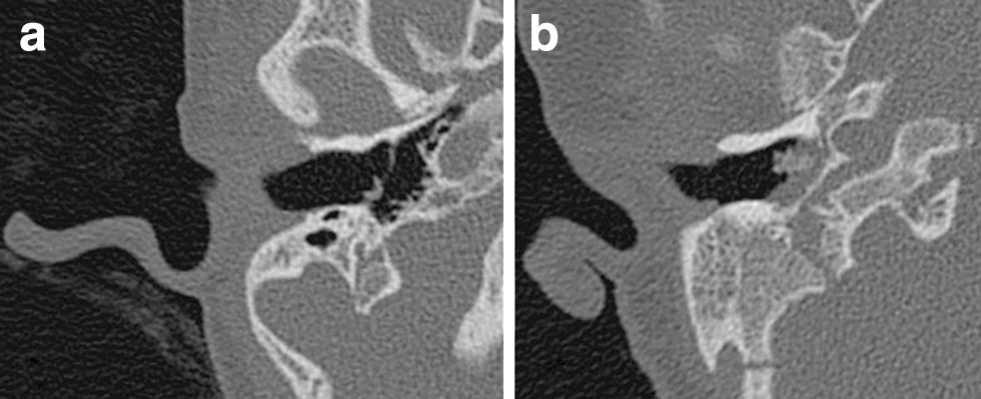
Mastoid size—axial CT images of a small mastoid (**a**) and a wide mastoid (**b**); both ears had a grommet in situ

Table 7 summarizes the observed anomalies expecting to be challenging in cochlear implant surgery. Figure 7 illustrates the differences in mastoid size between an ear with a small mastoid and an ear with a wide mastoid (AP-size).

### Phenotypes

No typical CHARGE phenotype of the temporal bone, i.e. a constant combination of several anomalies, could be determined. Some combinations of anomalies which were often seen are presented in Table 8. More than two-thirds of the patients (68 %) had an aberrant course of the vestibular aqueduct, and more than two-thirds (77 %) had aplasia of the SCC.

### Genotypes (Table 4)

The results of *CHD7* analysis were available for all 42 patients. We had 25 patients (50 ears, 59.5 % of 84 ears) with a truncating mutation (of which were 56 % nonsense, 20 % frameshift, 4 % deletions) and 15 patients (30 ears, 35.7 % of 84 ears) with a non-truncating mutation (47 % missense, 53 % splice site). In one patient an unclassified *CHD7* variant was detected (UV-missense) and in another patient no *CHD7* mutation was found. Remarkably, 12/42 patients did not fully comply with the clinical diagnostic criteria [2]. Of these 12 patients, eight had a non-truncating mutation, in one patient no mutation was found, and in another only an unclassified variant could be detected in *CHD7.* Thus, only 2/12 atypical patients (16.7 %) had a truncating mutation. In contrast, truncating mutations were found in 23/30 patients (76.7 %) who had clinically typical CHARGE syndrome.

Because no constant combination of anomalies could be identified, no correlation could be made between phenotype and genotype. Nevertheless, of the surgical challenging anomalies, SCC aplasia (Table 6) and oval window atresia (truncating 72 %, *P* = 0.001) were found more frequently in patients with truncating mutations than in those with non-truncating mutations (*P* < 0.05) (Chi-squared test). Cochlear anomalies (Table 5), petrosquamosal sinus (73 % truncating, *P* = 0.679) and an aberrant course of the tympanic portion of the facial nerve (67 % truncating, *P* = 0.602) were also found more frequently in patients with truncating mutations than in those with non-truncating mutations, but these results were not significant (Chi-squared test).

The patient without anomalies of the SCC, cochlea and windows, had a non-truncating mutation. In contrast, in the group with truncating mutations, there were no patients without anomalies of at least one of these structures.

## Discussion

Analysis of the available imaging material and genetic information of the Dutch cohort of patients with CHARGE syndrome revealed a great variability in anomalies of the temporal bone with possible implications for cochlear implantation. More anomalies were found in patients with truncating *CHD7* mutations than in those with non-truncating mutations. A shortcoming of this study is the variability in image quality leading to missing values of several fine anatomical structures (the imaging data were collected from different hospitals). Nevertheless, we were able to analyse the temporal bone and the anomalies, and to assess the potential impact on plans for cochlear implant surgery.

Temporal bone anomalies detected by CT are important when planning an operation. Vascular variations, missing anatomical landmarks such as the lateral semicircular canal or the vestibular system, an aberrant course of the facial nerve, or stenosis of the round window may hamper safe surgical access to the round window. Given our findings, when planning CI or ear surgery, care must be taken with regard to the reduced development of the mastoid, leading to a smaller access to the middle ear, especially in young children. In these cases, an endaural approach instead of a mastoidectomy [7], or a temporary intra-operative removal or anterior displacement of the posterior wall of the outer ear canal could be considered. Vascular anomalies could also complicate a mastoidectomy, since these may cause uncontrollable bleeding during surgery or postoperative thrombosis of the sigmoid sinus [8–10]. In our group of CHARGE patients, large emissary veins and a persistent petrosquamosal sinus were often present. Whereas emissary veins through the temporal squama are a common anatomical variant [11, 12] and easily dealt with during surgery, a persistent petrosquamosal sinus is rare in the general population (Koesling et al. [11] estimated this at 1 %). However, several authors have reported it to be present in 11–89 % of CHARGE patients [9, 13–15]. The highest incidence was described by Giesemann et al. [14], in patients who all had aplasia of the SCCs. In our patient population, which included patients with partially and fully developed vestibular systems, a persistent petrosquamosal sinus was seen in 13 %. The persistent petrosquamosal sinus can impede the surgical approach, this can be a reason to choose the contra lateral ear for CI.

SCC aplasia is a hallmark of CHARGE syndrome. *CHD7* is highly expressed in the developing ear and is required for development of the SCCs. Delayed fusion and altered gene expression contribute to SCC defects in *CHD7*-deficient mice [16]. Currently, the presence of SCC abnormalities is considered an important indication for performing sequencing of the *CHD7* gene and diagnosis [17]. In our study, we found that normal SCC were present in only one patient. However, during mastoidectomy, the lateral SCC serves as an important anatomical landmark, so the appearance of the SCCs, ranging from complete absence of all canals to normal development, should be meticulously described [7, 13]. In case of a lateral SCC aplasia, the tegmen serves as a paramount marker to direct the surgeon towards the antrum. Anomalies of the SCCs were associated with hypoplasia of the vestibule and a shortened vestibular aqueduct coursing straight to the posterior fossa. This confirms what was reported by Morimoto et al.: ‘An aberrant course of the vestibular aqueduct is hypothesized to be the result of semicircular aplasia and the associated displacement of normal surrounding structures’ [9].

The facial nerve is another structure at risk during cochlear implantation. As described in the literature, the facial nerve often showed an aberrant course in its tympanic or mastoidal portion [9, 13, 18]. The more medial course of the mastoidal portion of the facial nerve allows a surgeon to create a wider entrance to the middle ear (through a posterior tympanotomy). However, the aberrant course of the tympanic segment of the facial nerve, in particular when it covers the round window, may complicate creating the cochleostomy for intracochlear insertion of the cochlear implant. The aberrant facial nerve may be at risk of injury during cochleostomy [19] or may even be a reason to abort the implantation [20]. The association we observed of an aberrant course of the facial nerve with dysplastic stapes and absence of the oval window was described by Zeifer et al. in different aetiologies without CHARGE syndrome [21].

Absence or stenosis of the oval window was present in more than two-thirds of our patients and is a well-known feature in CHARGE syndrome [9, 13, 22, 23]. Stenosis or absence of the round window was seen less often (as confirmed in the literature). Yet this poses an additional challenge for the surgeon in choosing the optimal site for a cochleostomy.

The size and shape of the cochlea will influence the choice of CI-type. According to the literature, the cochlea is dysplastic in between 20 and 100 % of the ears described in patients with CHARGE syndrome [9, 18, 24, 25]. The anomalies vary from a fused second and apical turn to a cochlea with 1.5 turns [26–28]. We describe several cases with a shortened cochlea despite the presence of a basal, second and apical turn (referred to as hypoplasia type IV). To the best of our knowledge, this cochlear appearance has not been described in CHARGE syndrome before, but it appears to be consistent with the description of cochlear hypoplasia type IV in a recently published study by Sennaroglu [29] or may be comparable with the flattened cochlea observed by Elmaleh et al. [30] in patients with Waardenburg syndrome. However, the other temporal bone anomalies described in Waardenburg syndrome, besides SCC aplasia and the flattened cochlea, differ from our findings.

Both this cochlear anomaly, as well as the IPII and hypoplasia type III found in this study, should not cause any problems for the insertion of an electrode array as opposed to more severe malformations [31, 32]. The successful outcome of a cochlear implantation also depends on the presence of the cochleo-vestibular nerve.

In our phenotype-genotype analysis we showed that total aplasia of the SCC and oval window aplasia is more common in patients with truncating mutations than in those with non-truncating mutations—in agreement with the results of Corsten-Janssen et al. showing more anomalies in patients with truncating mutations [33]. Remarkably, the distribution of mutations present in our cohort differs from that reported for large cohorts in the literature. Our percentage of patients with non-truncating mutations (splice-site and missense) was relatively high with 35.7 % in comparison to Zentner et al. [5] and to Janssen et al. [4] who reported 23 and 20 % of patients with non-truncating mutations, respectively. This discrepancy might be because a CT is often used in mildly affected patients to check the semicircular canals and to provide further proof for the clinical diagnosis. Our cohort might be enriched with more mildly affected patients (12/42), and thus of missense mutations.

In general, we conclude that temporal bone findings in patients with CHARGE syndrome vary widely and should therefore be studied meticulously before performing any surgery. Imaging may exclude patients from cochlear implantation or reveal an aberrant course of the facial nerve, vascular and middle ear abnormalities that could complicate CI surgery. Such information is valuable and should be combined with records on the developmental and behavioural problems that are also common in CHARGE syndrome. Moreover, patients with CHARGE syndrome often have post-surgical complications due to their neurological and anatomical abnormalities [34]. A balance between the benefit of CI, the surgical procedure’s chance of success, and the anaesthetic risks should be sought by a multi-disciplinary team working with the patient and his/her family.

## Appendix

See Tables 9 and 10.
